# Assessment of Sit-to-Stand Transfers during Daily Life Using an Accelerometer on the Lower Back

**DOI:** 10.3390/s20226618

**Published:** 2020-11-19

**Authors:** Lukas Adamowicz, F. Isik Karahanoglu, Christopher Cicalo, Hao Zhang, Charmaine Demanuele, Mar Santamaria, Xuemei Cai, Shyamal Patel

**Affiliations:** Pfizer, Inc., Cambridge, MA 02139, USA; lukas.adamowicz@pfizer.com (L.A.); FikretIsik.Karahanoglu@pfizer.com (F.I.K.); Chris.Cicalo@pfizer.com (C.C.); Hao.Zhang2@pfizer.com (H.Z.); charmaine.demanuele@pfizer.com (C.D.); mar.santamaria@pfizer.com (M.S.); Xuemei.Cai@pfizer.com (X.C.)

**Keywords:** accelerometer, wearable technology, algorithm, free-living, sit-to-stand

## Abstract

The ability to perform sit-to-stand (STS) transfers has a significant impact on the functional mobility of an individual. Wearable technology has the potential to enable the objective, long-term monitoring of STS transfers during daily life. However, despite several recent efforts, most algorithms for detecting STS transfers rely on multiple sensing modalities or device locations and have predominantly been used for assessment during the performance of prescribed tasks in a lab setting. A novel wavelet-based algorithm for detecting STS transfers from data recorded using an accelerometer on the lower back is presented herein. The proposed algorithm is independent of device orientation and was validated on data captured in the lab from younger and older healthy adults as well as in people with Parkinson’s disease (PwPD). The algorithm was then used for processing data captured in free-living conditions to assess the ability of multiple features extracted from STS transfers to detect age-related group differences and assess the impact of monitoring duration on the reliability of measurements. The results show that performance of the proposed algorithm was comparable or significantly better than that of a commercially available system (precision: 0.990 vs. 0.868 in healthy adults) and a previously published algorithm (precision: 0.988 vs. 0.643 in persons with Parkinson’s disease). Moreover, features extracted from STS transfers at home were able to detect age-related group differences at a higher level of significance compared to data captured in the lab during the performance of prescribed tasks. Finally, simulation results showed that a monitoring duration of 3 days was sufficient to achieve good reliability for measurement of STS features. These results point towards the feasibility of using a single accelerometer on the lower back for detection and assessment of STS transfers during daily life. Future work in different patient populations is needed to evaluate the performance of the proposed algorithm, as well as assess the sensitivity and reliability of the STS features.

## 1. Introduction

The ability to perform a sit-to-stand (STS) transfer is altered due to aging as well as impairments associated with conditions like knee osteoarthritis, Parkinson’s disease, stroke, sarcopenia, and multiple sclerosis [1,2]. The performance of an STS transfer requires lower-limb muscles to generate forces to accelerate the body’s mass through a distance against the pull of gravity [3]. In addition to strength, factors such as balance, sensation and psychological state may also influence the performance of an STS transfer [4].

The clinical assessment of STS transfers can provide valuable information for tracking disease progression and assessing the efficacy of interventions. STS transfers are a key component of many widely used, performance tests like the 5-times (5x) sit-to-stand test, 30-s chair stand test, timed up and go (TUG), Tinetti performance oriented mobility assessment (POMA), Berg balance test and Egress test that are routinely used for assessing mobility [5]. Information captured during these assessments can help clinicians identify deficits (e.g., strength, balance), risk-factors (e.g., fall risk [6,7]), disease progression, and possible treatment effects.

Despite the valuable information that these mobility assessments can provide, their utility is limited as they are only administered episodically in the clinic. In addition, most performance-based assessment tests rely on either time or number of repetitions as an outcome measure, which limits the assessment to a single dimension and can fail to provide a comprehensive view of functional ability. Recent research has shown that while there is an association between measures of physical activity and physical performance, they seem to capture separate aspects of physical function [8,9]. Similarly, it has been reported that gait assessments performed in the lab are able to capture only a small portion of the variance observed in the real world [10,11]. Performance tests are typically designed to assess maximum capacity in a controlled setting, whereas physical activity in the real world occurs in the presence of cognitive and environmental challenges [12].

Advances in wearable sensing technology have led to a growing interest in the objective, real-world measurement of health and well-being [13,14]. Inertial measurement units (IMUs) are particularly well-suited for continuous measurement of movement as they can be packaged into small, power efficient devices that can be discretely worn at various locations on the body. Recent research efforts have shown that IMUs can be used for detection and assessment of STS transfers. Approaches that rely on multiple IMUs across several body locations [15,16] can detect STS transfers and measure STS kinematics [17,18] with relatively high accuracy. However, the poor wearability and high patient burden associated with the use of these multi-sensor systems make them ill-suited for long-term monitoring in free-living conditions and limits their application in clinical research. To address these limitations, several attempts have been made to use a single IMU located somewhere on the torso (typical locations: chest, waist, lower back) [19,20,21,22,23,24,25,26,27,28] or thigh/upper leg [28,29] for the detection and assessment of STS transfers. While the thigh-based approaches can be relatively robust because STS transfers can be detected by simply tracking orientation using an accelerometer, this device location is not ideal for measuring other aspects of mobility such as gait and balance. Torso-based approaches need to be more sophisticated because there is a high risk of false positives and false negatives. Most of the torso-based approaches rely either on using multiple sensing modalities (e.g., accelerometers, gyroscopes, barometric pressure) [20,21,22,25,26,27] or perform indirect detection of STS transfers [19,23,24]. While using gyroscopes can help improve accuracy [17], they consume significantly more power compared to an accelerometer [30] and can thus negatively impact the recording duration of a device. As a result, the devices would need to be bulky to accommodate a larger battery or require frequent recharging, which may have a negative impact on compliance with wearing the device. Indirect approaches can simplify the problem at the cost of limiting the detection of STS transfers to specific activity transitions (e.g., sit-to-walk). Therefore, a robust approach for the detection and assessment of STS transfers that relies on a single power-efficient sensing modality like an accelerometer would be extremely attractive for long-term monitoring and warrants further research.

While most of the work to date has been limited to the assessment of prescribed STS transfers performed in the lab, some recent efforts [23,24] have analyzed data captured under free-living conditions. Results show that features of STS transfers derived from IMU data captured during free-living conditions are able to detect differences related to age (e.g., young vs. old [23]), fall risk (e.g., healthy elderly vs. elderly with a history of falls [23]), and disease severity (e.g., mild Parkinson’s disease (PD) vs. severe PD [24]). In addition, multi-parametric approaches [16,19,20,23,24,26] relying on features that capture aspects associated with STS transfer quality (e.g., smoothness, variability, peak acceleration or velocity) during a STS transfer have been shown to be more sensitive for detecting clinically meaningful differences compared to approaches that rely only on duration [21,27,31]. More remarkably, these efforts [23,24] have provided initial evidence that assessment of STS transfers performed during free-living conditions help improve the detection of clinically meaningful differences compared to assessments performed in the lab. Further work is therefore required to understand the relationship between sensor-derived STS features captured during daily life and their ability to detect clinically meaningful differences, as well as assess the impact of monitoring duration.

The work presented herein focuses on the development and validation of an orientation-independent algorithm for detecting STS transfers using data from a single 3-axis accelerometer on the lower back. Data collected in the lab from healthy adults (younger and older) and Parkinson’s diease (PwPD) were used for validation. Performance of the proposed algorithm was compared with that of a commercially available multi-sensor system (APDM mobility lab, APDM wearable technologies) as well as a published algorithm [27] that was previously validated in PwPD. The proposed algorithm was then applied to data collected in free-living conditions from healthy adults (young and old) with the goal of assessing the ability of STS features to detect age-related group differences. Finally, simulations were performed to assess the impact of monitoring duration on the reliability of STS features.

## 2. Methods

### 2.1. Measurement Protocol

Data collected across two clinical studies were used for validation and testing of the STS detection algorithm.

#### 2.1.1. Healthy Adults Study

Sixty-six subjects were recruited in the greater Boston area, MA, USA (N=33 (17 Males) ages 65–85, N=33 (16 Males) ages 23–39; Inclusion criteria: no significant health issues, BMI between 18.5 and 30 $\frac{\mathrm{kg}}{m^{2}}$ or absolute weight <125 kg; Exclusion criteria: self-reported medical condition, recreational drug use, or medication use preventing study task completion, Vulnerable Elders Survey [32] total score > 3 including 0 in all activities of daily living (ADLs)) completed two in-lab visits spaced 7–14 days apart. One male subject in the older adults group was excluded as it was determined that he did not meet the inclusion criteria after study completion. Each subject performed various clinical assessments of mobility and health, including a 5x STS task using the same chair for all participants. Following the 5x STS task, subjects would also stand up normally in preparation for the following assessment, and this 6th STS transfer was included in the processing and analysis. During the 5x STS task, subjects sat on a rigid chair and were instructed to fold their arms across their chest and perform five consecutive STS as fast as they can. However, the 6th STS was performed independently by the subject with no instruction other than where to go for the next task. During the 7–14 days between lab visits, subjects wore an accelerometer on their lower back (lumbar region) and a wrist sensor on their non-dominant wrist while going about their daily lives (all subjects were community dwelling). Only the lumbar sensor data and STS assessment are presented in this work. The final protocol and informed consent documentation were reviewed and approved by Advarra Institutional Review Board (study ID: Pro00029419). All participants gave written consent prior to enrollment.

#### 2.1.2. PD Study

Thirty-five subjects were recruited in the greater Boston area, MA, USA (N=35 (23 Males), ages 46–79 [68.31±8.03 years], Hoehn and Yahr stage I/II/III: 2/26/7; Movement Disorder Society-Unified Parkinson’s Disease Rating Scale (MDS-UPDRS) III: 52.86±16.03; Inclusion criteria: clinical diagnosis of PD, on L-dopa therapy, able to recognize wearing-off periods, with Hoehn and Yahr stage ≤3; Exclusion criteria: presence of other comorbidities (e.g., head injuries, psychiatric illness, cardiac disorders), recent treatment with investigational drugs, pregnant women, allergy to silicone or adhesives). PwPD participated in the study involving two in-clinic visits. Participants were randomly assigned to be in ON state (i.e., well controlled motor symptoms, approximately 1 h after medication intake, confirmed by patient self-report and clinician report) in one visit and OFF state (i.e., poorly controlled motor symptoms, at least 3 h after medication intake, confirmed by patient self-report and clinician report) in the other visit. These motor states were used to assess symptom severity and motor performance during the two extremes of a motor fluctuation cycle. During each visit, subjects performed a battery of motor (including STS transfers) and non-motor tasks associated with clinical assessments, as well as activities of daily living (e.g., putting on a coat, drinking water from a cup, folding laundry). During the course of each visit, subjects performed a mix of scripted (5x STS task) and natural STS transfers using the same chair for all participants. The natural STS transfers consisted primarily of several isolated single transfers performed before the 5x STS task and a few transfers after the 5x STS task. The motivation behind including data from PwPD in this study was to evaluate the performance of STS detection methods in an impaired population. The PD study had approval from Tufts Medical Center and Tufts University Health Sciences Institutional Review Board (study ID: 12371). All participants provided written informed consent prior to enrollment. A complete description of the participant characteristics can be found elsewhere [33].

### 2.2. Instrumentation

During the in-lab portion of the healthy adults study and PD study, all subjects wore 6 IMU devices (Opal v2, APDM, Portland, OR, USA) across their body. The devices were attached using straps to both feet and wrists, sternum and lower back (approximately near the fifth lumbar vertebrae). The IMUs were set to sample at 128 Hz.

During the in-lab portion, subjects who participated in the healthy adults study were recorded with an 8-camera, markerless optical motion capture system (Simi Shape 3D, Simi Reality Motion Systems, Unterschleissheim, Germany) with a frame rate of 100 Hz.

During at-home portion of the healthy adults study, subjects wore two devices (GENEActiv, Activinsights, Kimbolton, UK): one on their non-dominant wrist and another on the lower back. Devices recorded 3-axis accelerometer data (range: ±8 g, sampling rate: 50 Hz) and were attached to the body using straps. Data were stored locally on the device and downloaded for offline processing following the return of the device to the study site.

Subjects in the PD study were also recorded using a depth sensing video camera (Microsoft Kinect, Microsoft, Redmond, WA, USA). The 2D video recordings were used by trained reviewers to generate activity annotations.

IMUs were approximately aligned with anatomical axes (anterior-posterior, medial-lateral, and vertical), however the proposed method is orientation independent, and does not rely on this configuration. While subjects had IMUs attached to other body locations, only data recorded from the device located on the lower back were considered in this work. For both studies, no additional calibration was performed on the sensor output other than that performed by the default manufacturer settings.

### 2.3. Ground Truth Methods

Two different ground truth methods were used, based on what data were available in each study. The Healthy Adults study used motion capture data as ground truth, while the PD study used the video recordings as ground truth.

#### 2.3.1. Motion Capture Method

A markerless optical motion capture method (Simi Shape 3D, Simi Reality Motion Systems, Germany; 100 Hz sampling rate) was considered as ground truth for the in-lab portion of healthy adults study. Synchronization between the IMU and Simi data was achieved by applying an offset, which was calculated by measuring the lag associated with the peak of the cross-correlation function between measured (IMU) and calculated (Simi) acceleration of the right foot during three foot stomps (time synchronization event in the protocol). A region of interest was manually selected to include the 5x STS task as well as a sixth STS transfer that was performed naturally following the 5x task by all participants. Right shoulder position signal (L2 norm of X, Y and Z) was then used to locate STS transfers by finding the local minima and maxima in the region of interest to indicate the beginning and end, respectively.

#### 2.3.2. Video Method

STS transfers annotated using trained human raters manually reviewing 2D video recordings (Microsoft Kinect, Microsoft, Redmond, WA, USA) were used as ground truth for the PD study. Two raters reviewed each video recording and identified the start and end times of each behavior of interest (e.g., walking, sit-to-stand, turning) observed during the course of a visit. Later, an arbitrator reviewed both set of ratings and resolved any inter-rater disagreements (e.g., different start times). A detailed description of the annotation process has been published previously [34]. To synchronize data between IMUs and Kinect video, an offset computed by cross-correlating the acceleration signal at the sternum measured by the IMU device and Kinect camera (second derivative of the displacement of the sternum key point) during the performance of a STS transfer was applied.

### 2.4. Reference Methods

#### 2.4.1. APDM Method

In-lab data collected in the healthy adults study during the 5x STS task were processed using the manufacturer’s proprietary algorithm (‘APDM’ method). This method relies on data (acceleration and angular velocity) from two IMU devices located on the trunk (sternum and lower back) to detect individual STS transfers. This method was chosen as a reference for the in-lab data as it has been specifically developed to detect STS transfers performed during the 5x STS task.

#### 2.4.2. AGR Method

A previously published method (‘Acceleration-Gyroscope Reference (AGR)’ method) [27] was implemented for comparing the performance of the proposed method. The AGR method was selected for comparison because it was originally validated in a PwPD population, and was developed for detecting STS transfers from data recorded under free-living conditions. The AGR method was only applied to the test group from the PD study, and its performance was evaluated relative to the video annotations. While both the proposed and AGR methods use signal processing and heuristics for the STS detection, the proposed method requires only acceleration data, while the AGR method requires acceleration and angular velocity data.

### 2.5. Proposed Method

The proposed method relies only on 3-axis accelerometer data and works in any device orientation, which removes the burden on the end user to wear the device in a specific orientation or perform a calibration step each time the device is worn. The proposed method (‘displacement’ method) can be split into two distinct parts: (1) acceleration filtering with potential STS identification, and (2) confirmation of the STS transfer. The first step applies several filters and transformations to the raw acceleration signal and locates periods were an STS transfer may have occurred. The second step takes the possible transfers, and either confirms that a transfer did occur, or rejects it. A constraint requiring stillness before a transfer was added as an option to the displacement method, yielding a variation of the algorithm that will be referred to as the ‘stillness+displacement’ method. While the ’displacement’ method is well-suited for prescribed tasks (e.g., 5x STS), the ’stillness+displacement’ method is more appropriate for daily life as it assumes STS transfers will be preceded by a period of stillness.

Algorithm 1 shows pseudo-code of the algorithm for filtering and locating possible STS transfers. Vector magnitude (*a*) of the measured acceleration ($y_{acc}$) signal is first zero-phase filtered using a fourth order Butterworth filter with a cutoff frequency of 5 Hz to remove high frequency components. The resulting signal is smoothed by taking a rolling mean (RM) with a 0.25 s window with equal weighting, yielding the ‘RM’ acceleration. A continuous wavelet transform (CWT) is applied to the RM acceleration, with coefficients in the 0–0.5 Hz band summed at each time point to yield the CWT ‘power’. Peaks in the CWT power with a minimum time difference of one second, and a minimum amplitude greater than the standard deviation of the power are identified as possible STS transfer time points.
**Algorithm 1.** Raw acceleration filtering and transformations, and STS possible location identification. RM is rolling mean, CWT is continuous wavelet transform.**Data**: $y_{acc}=\left[y_{ax},y_{ay},y_{az}\right]$ **Result**: *a*, $a_{filtered}$, $a_{RM}$, *P*, $STS_{possible}$ $a\;\;\leftarrow \;\;\sqrt{y_{ax}^{2}+y_{ay}^{2}+y_{az}^{2}}$; $a_{filtered}\;\;\leftarrow \;\;butterworth\left(|a|,order=4,cutoff=5\;\mathrm{Hz}\right)$; $a_{RM}\;\;\leftarrow \;\;RM\left(a_{filtered},window=0.25\;s\right)$; $c\left(F\right),F\;\;\leftarrow \;\;CWT\left(a_{RM},gaus1,scale=1\to 64\right)$; $P\;\;\leftarrow \;\;\sum_{f=0\;\mathrm{Hz}}^{0.5\;\mathrm{Hz}}c_{i}\left(f\right)$; $STS_{possible}\;\;\leftarrow \;\;peaks\left(P,height=\sigma_{P},distance=1\;s\right)$;

Once the possible time points of STS transfers are identified, they can be confirmed or discarded using the algorithm shown (as pseudo-code) in Algorithm 2. The periods of stillness are first found using rolling means and standard deviations (SDs, rolling SD: RSD) of the filtered acceleration and jerk (time derivative of filtered acceleration) signals. At any time point where the filtered acceleration RM and RSD (window: 0.3s) are less than 0.15 $\frac{m}{s^{2}}$ and 0.1 $\frac{m}{s^{2}}$ respectively, and the jerk RM and RSD (window: 0.3s) are less than 2.5 $\frac{m}{s^{3}}$ and 3.0 $\frac{m}{s^{3}}$ respectively, are considered to be still. Beginnings and ends of still periods longer than 0.3s are then found.

In order to get an estimate of the vertical component of acceleration, the raw acceleration is zero-phase filtered using a fourth order Butterworth filter with a cutoff of 0.8 Hz, and normalized to yield a unit vector in the direction of gravity. The dot product of raw acceleration and this estimate is taken to give the vertical acceleration, per

(1) $$\begin{array}{cc} a_{vert} & =a\cdot \hat{g} \end{array}$$

where $a_{vert}$ is the vertical acceleration, *a* is the raw (measured) acceleration and $\hat{g}$ is the estimate of the direction of gravity.

Each possible STS transfer is then evaluated against a predefined set of conditions and discarded if any of the conditions are not met. First, the region of integration for the vertical acceleration is determined. If stillness is required (‘stillness+displacement’ method), the beginning of the integration region is the end of the stillness period located within the 2s preceding the STS location being validated. The end of the integration region is defined as the start of the next stillness period longer than 0.3s and within 30s after the STS location. If this condition is not met, STS location plus 5s is used as the end of the integration region. The vertical acceleration is then double integrated over the integration region to derive vertical velocity (first integral) and displacement (second integral). If the integration region ends with the accelerometer being still, the slope between the first and last points is removed, per

(2) $$\begin{array}{cc} v_{vert} & =\int_{t_{0}}^{t_{N}}a_{vert}dt \end{array}$$

(3) $$\begin{array}{cc} v_{vert} & =v_{vert}-\frac{v_{\left(vert,N\right)}-v_{\left(vert,0\right)}}{t_{N}-t_{0}}t \end{array}$$

where $v_{vert}$ is the velocity in the vertical direction, $t_{0}$ is the integration region start, and $t_{N}$ is the integration region end, and *t* indicates time. If the accelerometer at the end of the integration region is not still, then the velocity is detrended using the best fit slope across all of $v_{vert}$. No modification is applied to displacement.
**Algorithm 2.** Algorithm for validating sit-to-stand (STS) transfers given the filtered acceleration and the possible STS locations from the first processing step. RStats is the computation of the rolling mean and standard deviation.
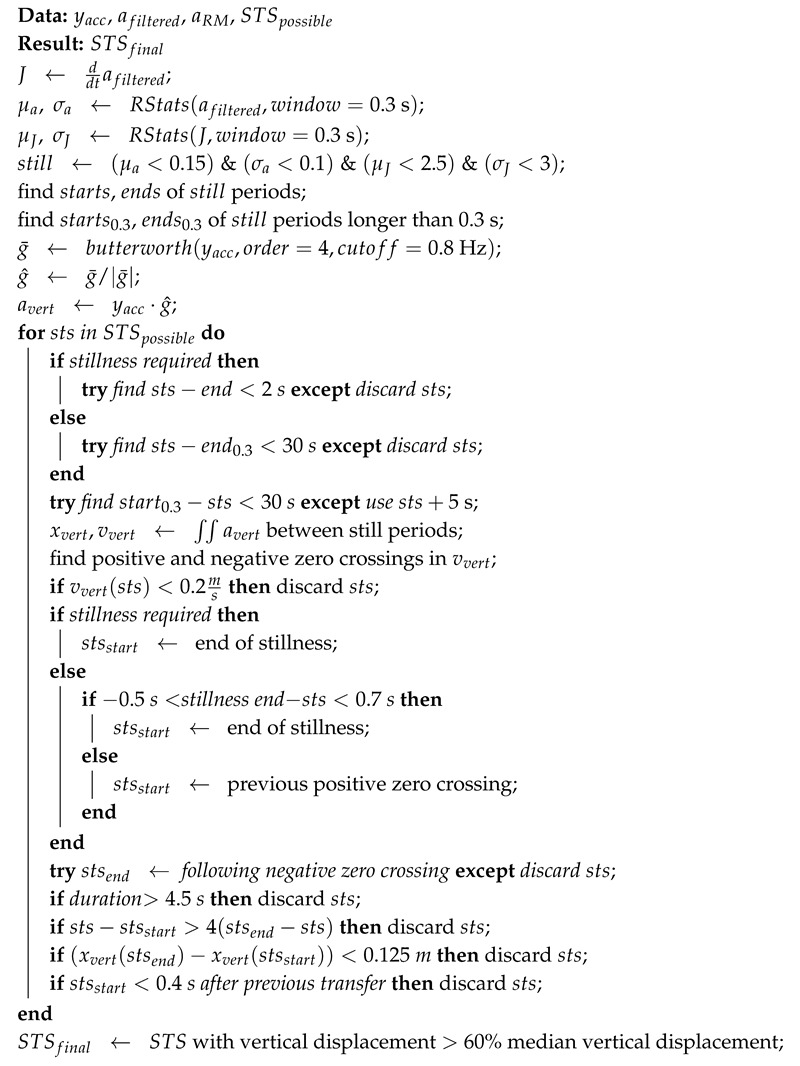


With the vertical velocity and displacement computed, the beginning and end of the STS transfer can be determined, and transition validity checks performed. The first check is to ensure that the vertical velocity during the STS transfer exceeds 0.2 $\frac{m}{s}$. Next, the beginning of the STS transfer is found. For the displacement method, the previous positive zero crossing is used as start time of the transfer, unless the end of a still period is within −0.5s to 0.7s of the STS time point. The displacement + stillness method takes the end of the preceding stillness period as start time of the transfer. Both methods (’displacement’ and ’displacement + stillness’) define the end of the transfer as the negative zero crossing of the vertical velocity signal following the STS location. The STS is discarded if this condition does not occur. The following quality checks are then performed:

1. based on prior work, STS duration must be less than 4.5s [20,31,35],
2. portion of the STS transfer occurring before the STS time point must be less than 4 times as long as the portion of the transfer occurring after it,
3. vertical displacement must be more than 0.125m, which is based on the assumption that a typical adult rising from an upright chair should experience a vertical displacement comparable to upper leg length (male: >0.397 ± 0.0024 m, female: >0.358 ± 0.0022 m [36]), and accounting for integration errors, and
4. the duration between consecutive STS transfers cannot be less than 0.4s, which is based on the impossibility of performing two STS transfers without a stand-to-sit transfer between them.

**Figure 1:**
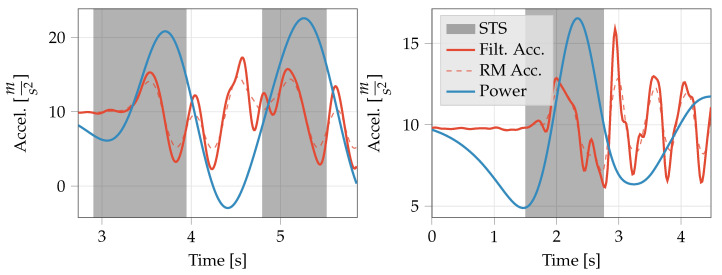
Example acceleration and processed signals showing the automatic segmentation of STS transfers by the proposed algorithm. (**Left**) Example in-lab signal from the 5x STS, with STS transfers detected with the displacement method. (**Right**) Example at-home signal with STS transfer detected with the displacement + stillness method. STS is sit-to-stand, Filt. Acc. is the filtered acceleration, RM is rolling mean, and Power is the Continuous Wavelet Transform power. Note: Power is not to scale.

As a final step, any accepted STS transfers with a vertical displacement less than 60% of the median of all detected transfers (per subject and per time series) were removed to eliminate partial transfers. The entire processes are described in Algorithm 2. Figure 1 shows examples transitions that were detected by the proposed algorithms in-lab and at-home. Note that for the at-home example, activity is present following the transition but is not identified as another STS transfer.

All processing was done offline using Python, and the source code is available in an open-source Python package called Sit2StandPy [37].

### 2.6. Validation

A training-validation split of ≈70–30% was randomly applied to the participants performing the in-clinic STS transfers, resulting in 19 validation participants for the healthy adults study. In order to have enough patients for testing, a ≈40–60% training-validation split was randomly applied to the PD study, resulting in 20 validation participants. Validation participant data were unseen until testing the final algorithm.

Validation was performed by comparing STS transfers detected by sensor-based methods (i.e., reference methods and proposed method) with ground truth annotations generated by the motion capture method for healthy adults study and the video method for PD study. To assess the performance of STS detection methods from a classification point of view, sensitivity and precision were computed as the primary interest was on reducing false positives while maintaining good sensitivity. Sensitivity was calculated as follows:

(4) $$\begin{array}{cc} \mathrm{Sensitivity} & =\frac{TP}{TP+FN} \end{array}$$

where TP is the number of true positives, and FN is the number of false negatives. Precision was calculated as follows:

(5) $$\begin{array}{cc} \mathrm{Precision} & =\frac{TP}{TP+FP} \end{array}$$

where FP is the number of false positives.

In addition, Jaccard index was computed to assess the overlap between the detected and ground truth STS transfers. The Jaccard index is a measure of the overlap between two regions (also called ‘intersection over union’). For two areas *A* and *B*, the index is defined as follows:

(6) $$\begin{array}{cc} J & =\frac{\left|A⋂B\right|}{\left|A⋃B\right|}=\frac{\left|A⋂B\right|}{\left|A|+|B|-|A⋂B\right|} \end{array}$$

where *J* is the Jaccard index. The Jaccard index is a normalized value between 0 and 1, with 1 indicating perfect overlap and 0 indicating no overlap. The mean Jaccard index was obtained for each visit, and then the mean and standard deviation across all subjects and visits were calculated.

The start and stop time deltas were defined per

(7) $$\begin{array}{cc} \mathrm{Start}\;\Delta & =t_{\left(start,pred\right)}-t_{\left(start,true\right)} \end{array}$$

(8) $$\begin{array}{cc} \mathrm{Stop}\;\Delta & =t_{\left(stop,pred\right)}-t_{\left(stop,true\right)} \end{array}$$

where Δ indicates a time difference, and $t_{pred}$ is a proposed method predicted time, and $t_{true}$ is the true time. All metrics were calculated for both the proposed and reference methods.

### 2.7. Analysis of Sit-to-Stand Features

The ability of STS features to differentiate between the younger and older cohorts in the healthy adults study was assessed. The ‘displacement’ method was applied to data collected in the lab whereas ‘displacement + stillness’ method was applied to data collected at home for detecting STS transfers. For at-home recordings, a day was defined from 8:00 to 20:00 based on our assumption that most of the daytime activities occur between this window. Subjects were not required to wear the devices while sleeping or bathing.

The following four features were derived to quantify each detected STS transfer: (1) duration (s), (2) maximum acceleration (g), (3) minimum acceleration (g), and (4) spectral arc length (SPARC, unit less) [38]. SPARC is a measure of signal smoothness with higher values indicating smoother movements, and is a quantification of a motion’s Fourier magnitude spectrum. For each subject, summary statistics for each feature were derived by calculating the median and coefficient of variation (CoV)

(9) $$\begin{array}{cc} \mathrm{CoV}(x) & =\frac{\sigma_{x}}{\mu_{x}} \end{array}$$

where σ is the standard deviation and μ is the mean. Data collected during the two in-lab visits in the healthy adults study were used to assess test-retest reliability of STS features using the intra-class correlation coefficient across all participants (ICC, computed for two-way random effects, absolute agreement, single rater/measurement) [39]. STS features from both in-lab and at-home were tested for their ability to discriminate between the younger (age 18–40) and older (age 65–85) cohorts, as well as discriminate between healthy young and old cohorts and PwPD in the ON and OFF state, using the following metrics: receiver operating characteristic area under the curve (AUC), Davies–Bouldin index (DBI) [40], and *p* values for age group obtained by mixed effect regression model with repeated measures (MMRM). AUC indicates the probability to correctly rank a random positive sample higher than a negative one, and the DBI assesses the separability of two clusters, with values below 1 indicating linear separability. The subset of healthy adults study in-lab data having two visits (60 subjects: 31 younger) was summarized by median and CoV accross all transitions in each visit and fed into MMRM as dependent variables; age group, sex, and visit were entered as independent variables, including their interactions; height and muscle mass were added as covariates, and subject as random effect. The at-home data were also summarized similarly across all transitions during the at-home monitoring period and fed into MMRM including age group and sex as main independent variables, their interactions, and height and muscle mass as covariates. The day type (weekdays vs. weekend) did not have any effect on the STS features and therefore was omitted from the analyses. The dependent variables were rank transformed in order to account for non-normality. MMRM analyses were followed by ANOVA, and all *p*-values were corrected for multiple comparisons using false discovery rate. The comparison between PwPD and healthy participants was performed using linear regression followed by ANOVA. The group (younger/older/PD ON/PD OFF), sex, and their interaction were fed as independent variables; height and weight were entered as covariates. Data from healthy subjects were averaged across the two visits. Pairwise comparisons were also performed to discriminate between the healthy and PD groups, multiple comparisons across the groups were corrected with Bonferroni correction.

The effect of at-home monitoring duration on the ability of STS features to detect group differences was assessed. The first six days with at least 10 STS transfers per day were identified for subjects in the healthy adults study. Days were then randomly sampled with replacement to form 1000 sets, each ranging from one to five days. STS features were computed for each set and compared to the reference feature values computed from all six days using two-way random effect, absolute agreement, single rater/measurement ICC. This resulted in 1000 ICC values per set. Minimum monitoring duration for each feature was determined by using a threshold of 0.7 for median ICC value [39]. When examined separately, similar results were observed for young and old subjects based on the 0.7 median ICC cutoff, and therefore only combined results are presented here.

Table 1 lists a high-level summary of the analysis performed on the different studies and sub-studies.

## 3. Results

### 3.1. Algorithm Validation (In-Lab Data)

For the healthy adults study, data from 19 subjects across 35 in-lab visits were in the validation group for the proposed method. Three visits were removed due to poor synchronization between signals from the wearable device and the SIMI system. Six STS transfers (five from 5x STS task and a following 6th natural transfer) were derived from each visit, resulting in a total of 210 STS transfers.

The displacement method achieved higher sensitivity (0.947) and precision (0.990) compared to the APDM method (reference), which was able to achieve sensitivity and precision of 0.863 and 0.868, respectively (Table 2) on the first five transitions. While the start and stop time differences for both methods were comparable, the Jaccard index for the displacement method (0.700±0.16) was slightly higher than the APDM method (0.645±0.16).

For the PD study, data from 33 visits across 20 subjects were in the validation group for the proposed method. Seven visits were removed due to synchronization issues between data from wearable devices and video recordings (which were used to generate the ground truth STS annotations). In addition, all ground truth STS annotations were inspected to ensure that partial transfers were excluded from the validation set. As a result, after excluding a total of 105 partial STS transfers, 314 complete STS transfers were available for validation.

The displacement method was able to achieve higher sensitivity (0.853 vs. 0.779) and precision (0.988 vs. 0.643) compared to the AGR method. As in the case of the healthy adults, the displacement method achieved a slightly higher Jaccard index compared to the AGR method. In addition, while the start time difference was higher (0.172±0.47s vs. 0.451±0.58s) for the AGR method, the stop time difference was lower (−0.182±0.62s vs. −0.016±0.46s).

**Figure 2:**
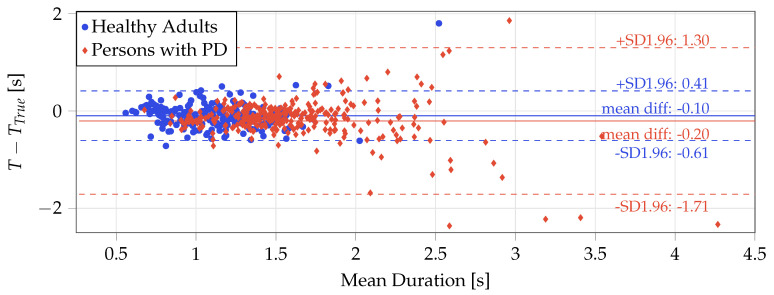
Bland–Altman plot showing difference in the duration of the STS transfers detected using the displacement method (*T*) and ground truth ($T_{true}$) for the in-lab data from the healthy subjects (red dots) and the persons with PD (blue diamonds). Two outliers in the PD data fall beyond the range of the graph limits and are therefore not visible.

Figure 2 shows the Bland–Altman plot of the STS duration derived using the displacement method and ground truth STS duration for the healthy adults study and PD study. While limits of agreement (95%) of −0.61s and 0.41s with a mean difference of −0.10s indicate good agreement for the healthy adults, a Wilcoxon signed ranks test indicated that the difference in STS duration between the two methods is significant (*p*-value ≪0.05). In the case of PwPD, while the mean difference is relatively low (−0.20s), the limits of agreement are significantly larger (−1.71s and 1.30s) compared to healthy adults. The mean difference is statistically significant (Wilcoxon signed ranks test, ≪0.05) and appears to be higher for slower STS transfers (mean duration >2 s). However, it is worth noting that all but one of the lower outliers (including two that are outside the graph limits) are from a single subject who had the worst clinician rating in the MDS-UPDRS for the chair stand task among subjects in the validation set.

### 3.2. Algorithm Application

The proposed method was then applied to in-lab and at-home data from the healthy adults study for detecting STS transfers and deriving features to assess quality of each detected transfer. On average, subjects performed 30.4±9.5 transitions per day, or 3.6±2.9 transitions per hour.

Table 3 provides summary statistics of the STS features derived at home and in the lab as well as ICC with lower and upper bounds for the 95% confidence intervals for STS features derived from the two lab visits. Median maximum acceleration and median minimum acceleration demonstrated moderate to good test-retest reliability [39]. Test-retest reliability was poor to good for median duration and median SPARC, poor to moderate for CoV of maximum acceleration and CoV of minimum acceleration, and poor for CoV of SPARC and CoV of duration [39].

The ability of features of STS transfers to detect differences between age groups (older and younger) as well as healthy and PwPD was also assessed. When comparing age-related group differences (young and old healthy adults), the direction of change for most STS features remained the same in the lab and at home, with the exception of CoV of maximum acceleration (0.197 to 0.206 at home, 0.048 to 0.042 in lab).

Table 4 shows the top five features (sorted by AUC) for each group-wise comparison. Moroever, 26 PwPD of 35 were used for this analysis, four were missing visit ON/OFF state information, one was missing annotations, and four more were missing age/weight/sex data. A total of six comparisons were made, five in-lab: PD ON state with healthy old subjects and healthy young subjects, PD OFF state with healthy old subjects and healthy young subjects, and healthy old subjects compared with healthy young subjects in-lab and at-home. The comparison between PwPD in the ON and OFF states was also made, but showed poor discriminative ability with all AUC values below 0.6 and DBI values above 4.3, and *p*-values of 1.0. One at-home comparison was made: healthy old subjects compared with healthy young subjects. In all comparisons of healthy adults with PwPD in both ON and OFF states, good discriminative ability was observed, with the top feature having an AUC above 0.85. The comparisons with younger subjects showed a larger difference, as expected. Unsurprisingly, when comparing healthy subjects (both young and old) to PwPD in the OFF state, the group differences were quite large, and in both cases, the top features had very low DBI values, indicating excellent separability.

When comparing old vs. young healthy subjects in-lab and at home, several STS features were able to discriminate between the two age groups from data collected in the lab as well as at home. However, the STS features derived from data collected at home had significantly higher discriminative power (AUC >0.8 for the top four features) compared to those derived from data collected in the lab. Interestingly, while median duration was the top ranked feature in the lab (and in the top 2 of ranked features in all in-lab comparisons between healthy participants and PwPD), it was not able to significantly differentiate between the older and younger groups of healthy subjects at home. From a clustering perspective comparing healthy old and young groups, the lowest DBI in the lab was 2.658 (median min. accel.) compared to 1.334 (CoV SPARC) for STS transfers recorded at home. *p* values (MMRM model) were lower at home, all well below α=0.05 except for median SPARC, while all were above α=0.18 for the in-lab features in the old-young comparison.

**Figure 3:**
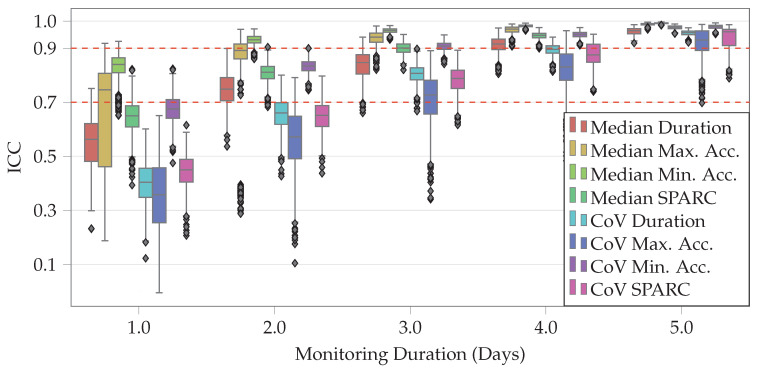
Comparison of STS features during random subsets of one to five days with STS features derived using six consecutive days across all healthy subject study participants. 0.7 and 0.9 cutoffs are marked as dashed lines. ICC was two-way random effects, absolute agreement, and single rater/measurement. From left to right, features are median duration, median of maximum acceleration, median of minimum acceleration, median SPARC, CoV of duration, CoV of maximum acceleration, CoV of minimum acceleration, and CoV of SPARC.

In order to assess the impact of monitoring duration on estimation of STS features, only subjects with at least six days of data at home and at least 10 STS transitions per day were included in the analysis. This resulted in the inclusion of 65 subjects (female: 33, old: 32) out of 65 who participated in the healthy adults study. Figure 3 shows the distribution of ICC values (with respect to all six days) for each of the STS features as we simulate increasing the monitoring duration from one day to five days. Cutoffs of 0.7 and 0.9 for median ICC value were used to determine good and excellent reliability, respectively [39]. At three days, the median ICC values for all eight STS features were above 0.7, and they were at or above 0.9 for four STS features (median SPARC, CoV of minimum acceleration, median of minimum acceleration and median of maximum acceleration). This indicates that a monitoring duration of three days might be sufficient for capturing sufficient number STS transfers to characterize their performance in this population.

## 4. Discussion

A method for detecting STS transfers using only acceleration measurements from the lumbar region was presented and validated using data collected in the lab from healthy adults and PwPD. The method was then applied to data collected in the lab and at home from healthy adults to assess if features of STS transfers were able to differentiate between two cohorts of young and old healthy adults. Validation results show that the proposed method achieved better performance compared to a previously published method, as well as a commercially available system. Furthermore, features derived from STS transfers captured at home were able to discriminate between the two age groups (see Table 4) at a higher level of significance compared to features derived from data collected in the lab during the performance of prescribed STS transfers. Finally, the analysis of data collected at home showed that a monitoring duration of three days is sufficient for capturing the variability observed over monitoring duration of six days. These results underscore the need for instrumented at-home assessment of STS transfers (in addition to other aspects of mobility like gait and balance). Such assessments can improve our understanding of functional ability in people with mobility limitations by enabling the capture of objective, high-resolution data across academic research, clinical trials and clinical practice. In clinical trials, this can translate into smaller sample sizes [41] and accelerate the development of therapies by improving our ability to assess the efficacy of new interventions. In clinical practice, longitudinal at-home monitoring (as opposed to episodic in-clinic assessments) may enable the early identification and treatment of deficits, resulting in improved outcomes.

On data collected in the lab during the healthy subjects study, the proposed method was able to achieve start and stop time differences comparable to the APDM method. However, both sensitivity (0.947) and precision (0.990) were higher for the proposed method compared to the APDM method (0.863 and 0.868, respectively). 95% limits of agreement of transfer duration of −0.61s and 0.41s also indicate good agreement with the ground truth annotations (Figure 2) in healthy adults. Overall, the proposed method demonstrated an improvement in performance over the APDM method.

Similar results were observed on data collected in the PD study. When compared to the AGR method, the proposed method was able to achieve better sensitivity (0.853 vs. 0.799), higher precision (0.988 vs. 0.643), higher Jaccard index (0.807 vs. 0.639) and smaller start time difference (0.172 vs. 0.451). These results, which can be attributed to the additional rejection criteria embedded in the algorithm, indicate that the proposed method will result in fewer false positives and be able to detect STS transfers with greater fidelity compared to the AGR method, despite relying only on data from a single 3-axis accelerometer. It is worth noting that the AGR method relies on data from a 6-axis IMU (accelerometer and gyroscope) and was previously validated on PwPD, achieving a sensitivity of 0.89 and 0.69 for PwPD without and with dyskinesia [27]. Future work should focus on evaluating the performance of the proposed algorithm in patient populations with different levels of impairment.

Comparing STS duration measured by the proposed method to expert annotations, a mean difference of 0.2s was observed, with limits of agreement of −1.71s and 1.30s (Figure 2). The larger limits of agreement were primarily driven by larger differences between the proposed method and the expert annotations for longer STS transfers. Some of the expert annotations were significantly longer (>5s) compared to STS duration of 2.63±0.21s in PwPD [42], 3.19±1.08s in patients with knee osteo-arthritis [35], and 4.08±1.21s in the frail elderly [20], which have been previously reported in the literature. The longer annotations can be attributed primarily to three factors: (1) multiple STS transfer attempts before the completion of the transfer, (2) very slow initiation marked by a slow trunk lean forward phase, and (3) very slow postural straightening at the end of a transfer. Further work is required to validate the proposed method in naturalistic environments (e.g., an instrumented home or apartment [43]) to properly characterize its performance characteristics.

The proposed method was then applied to data collected in the lab and at home in the healthy adults study and a set of features were extracted from each detected STS transfer to assess the quality of performance. Median values of all four features indicated better STS performance (shorter duration, higher maximum acceleration, lower minimum, larger SPARC) for younger subjects, both in the lab and at home. CoV of the STS features derived from at home data were generally higher (with the exception of maximum acceleration) for young adults, which might indicate that they are able to perform STS transfers at a broader range of speeds compared to older adults. In addition, the average values of STS features derived from home data were comparable with previous studies. Transfer duration for the older group (1.103±0.26s in lab, 1.744±0.14s at home) were comparable with those found previously in the lab setting for elderly subjects in a variety of chairs and speeds (2.00±0.40s [35], 1.62±2.54s [44], and 1.0 to 3.1s [31]). To the authors’ knowledge, this is also the first time that SPARC [38] has been used for assessing STS transfers. STS transfers performed in the lab were generally smoother (lower value of SPARC) compared to those performed at home for both groups and those performed by the younger group were smoother than the older group. However, despite the sensitivity of the proposed features like min/max acceleration and SPARC at detecting age-related group differences, they are difficult to interpret. In contrast, current clinical assessments are based on timed performance tests (e.g., 5x STS test), which are easy to use for clinical decision making. Therefore, further investigations will be necessary to evaluate the proposed (as well as additional) sensor-derived features, validate these features in impaired populations and establish normative ranges that can be used for clinical interpretation.

Test-retest reliability of STS features was assessed based on data collected in the healthy adults study during two lab visits. ICC values ranged from poor to good (0.0(−0.256−0.256) to 0.823(0.714−0.892)) [39], indicating that some metrics were more consistent than others. Potential reasons for this could be: (1) significant time interval between the two lab visits (7–14 days) causing deviation in subject-specific STS execution, and (2) insufficient number of STS transfers for reliable estimation of feature values in the lab. In fact, ICC was lower for CoV values (compared to ICC for median values) of all features except maximum acceleration.

Several STS features derived in-lab were able to discriminate between PwPD in both ON and OFF states and healthy young and old subjects. This was particularly true for the comparison between PwPD in the OFF state and healthy young, with AUC values above 0.89, DBI values below 1.1, and *p*-values <$10^{-5}$ for the top 3 features. In fact, features of STS transfers were similar or better at distinguishing between PwPD and healthy subjects than distinguishing between healthy old and young subjects. Interestingly, across three (out of four) of these comparisons (i.e., PwPD with healthy cohorts) the the top 3 features were the same (median minimum acceleration, median duration, and median SPARC—two of the features were present for the PD ON and healthy old cohort comparison). This ability to discriminate between healthy adults and PwPD is consistent with previous work using sit-to-walk transfers [24].

STS features derived from at-home data were able to discriminate between the two age groups at a much higher significance level (Table 4) compared to those derived from in-lab data. All five of the top features derived from at-home data were able to achieve a higher AUC (>0.675) than the top ranked feature (median duration) from in-lab data. Interestingly, while median duration was the best feature in the lab (consistent with prior works [45,46]), it was not among the top features at home. These results highlight a potential limitation of in-lab assessments like the 5x STS test, which require performance of the STS transfers as quickly as possible with arms crossed over the chest. In contrast, STS transfers captured at home are performed naturally and are therefore able to provide a more accurate picture of real-world functionalability. This observation is consistent with recent findings [10,11] that have shown that gait measurements performed in a lab setting are able to capture only a fraction of the variability observed in the home and community setting.

To assess the impact of at-home monitoring duration on STS features, simulations to test the reliability of STS features were performed by increasing the number of days of available data (Figure 3). All eight features were able to achieve a median ICC value higher than 0.7 (good reliability [39]) after three days and six features were able to achieve excellent reliability (median ICC >0.9) after four days, indicating that a monitoring duration of three to four days may be sufficient for assessing STS transfers in healthy adults. However, further investigations are needed to assess the impact of monitoring duration in populations with various types of impairments. In addition, only a maximum monitoring duration of six days was considered, which might not be sufficient to capture the broad range of variability in the performance of STS transfers at home.

## 5. Conclusions

A new method for detecting STS transfers using data from a lumbar-mounted accelerometer was developed and validated on healthy adults and PwPD. Features derived from sensor data were then assessed for their sensitivity to detect age-related differences during the performance of STS transfers in the lab and at home. Results show that the proposed method outperforms two reference methods, with slightly better performance than the proprietary APDM method in the healthy adults and significantly better performance than an IMU-based method in the PwPD. Features extracted from STS transfers detected at home were able to differentiate between the two age groups (younger and older) at a higher level of significance compared to STS transfers performed in the lab. These results contribute to the growing body of work highlighting the need for objective real-world assessment of mobility because performance tests administered in controlled settings like the lab or clinic are episodic and short, and hence are unable to capture the broad range of variability observed in the home and community setting. Simulations aimed at assessing the impact of monitoring duration on STS features measured at home showed that only three days of monitoring are sufficient to achieve good reliability in healthy adults. The work presented here lays a foundation for future investigations to validate the proposed method in patient populations and assess the ability of sensor-derived features of STS transfers at detecting clinically meaningful changes in their functional ability.

## Figures and Tables

**Table 1:** Summary of the type of analyses that were performed on datasets from different studies.

	Analysis	Healthy In-Lab	Healthy At-Home	PD In-Lab
STS Detection	Ground Truth Comparison	x		x
	Reference Method Comparison	x		x
STS Analysis	Test-Retest	x		
	Group Discrimination	x	x	x
	Minimum Monitoring Days		x	

**Table 2:** Performance of the proposed method on the validation set for the healthy subjects and PD study. Results are presented as mean (SD) where applicable.

Study	Method	Sensitivity	Precision	Avg. J ^1^	Start Δ [s]	Stop Δ [s]
Healthy Subjects	Displacement	0.947	0.990	0.700 (0.16)	−0.086 (0.21)	−0.187 (0.19)
[N = 19]	APDM ^2^ (Ref.)	0.863	0.868	0.645 (0.16)	−0.010 (0.24)	−0.199 (0.23)
PwPD	Displacement	0.853	0.988	0.807 (0.12)	0.172 (0.47)	−0.182 (0.62)
[N = 20]	AGR (Ref.)	0.779	0.643	0.639 (0.15)	0.451 (0.58)	−0.016 (0.46)

^1^ Jaccard Index, ^2^ APDM tested on only the 5x STS task, does not include the sixth extra transfer.

**Table 3:** Mean (SD) of features extracted from STS transfers from the two age groups (young and old) of healthy subjects, and an assessment of test-retest reliability based on the two in lab visits. Med. is median.

	Home ^1^ [N = 65]		Lab ^2^ [N = 60]		
Feature	Young	Old	Young	Old	ICC ^3^ (95% CI)
Median Duration [s]	1.711 (0.21)	1.744 (0.14)	0.963 (0.26)	1.103 (0.26)	0.510 (0.298–0.674)
Median Max. Acc. [$\frac{m}{s^{2}}$]	12.387 (0.56)	11.781 (0.39)	13.949 (2.22)	12.908 (0.69)	0.823 (0.714–0.892)
Median Min. Acc. [$\frac{m}{s^{2}}$]	7.550 (0.37)	8.037 (0.30)	4.780 (1.309)	5.509 (1.21)	0.748 (0.549–0.856)
Median SPARC	−2.299 (0.04)	−2.324 (0.03)	−2.032 (0.12)	−2.086 (0.12)	0.456 (0.231–0.635)
CoV Duration	0.382 (0.04)	0.363 (0.04)	0.164 (0.05)	0.162 (0.05)	0.000 (−0.256–0.256)
CoV Max. Acc.	0.197 (0.07)	0.206 (0.09)	0.038 (0.02)	0.035 (0.03)	0.501 (0.283–0.671)
CoV Min. Acc.	0.149 (0.03)	0.110 (0.02)	0.141 (0.10)	0.109 (0.06)	0.590 (0.395–0.735)
CoV SPARC	−0.078 (0.01)	−0.063 (0.01)	−0.051 (0.02)	−0.048 (0.02)	0.160 (−0.100–0.399)

^1^ Displacement + Stillness, ^2^ Displacement (N = 60 with 2 visits, 31 young), ^3^ 2-way random effects, absolute agreement, single rater/measurement.

**Table 4:** Top ^1^ five features with best performing STS metrics for assessing separation and classification of the compared participant groups. Comparison is based on all detected transfers per subject during the corresponding studies. All data are from in-lab, unless noted otherwise. In-lab data were from 26 PwPD, 29 healthy old participants, 31 healthy young participants using the proposed displacement method. At-home data were from 32 healthy old participants, 33 healthy young participants, using the proposed displacement + stillness method.

	Feature	AUC	DBI	*p*	Feature	AUC	DBI	*p*
**Comparison**	**PD ON & Healthy Old**				**PD ON & Healthy Young**			
	Med. Min. Accel.	0.850	1.560	<$10^{-5}$	Med. Min. Accel.	0.922	0.959	<$10^{-8}$
	Med. Duration	0.824	1.502	<$10^{-3}$	Med. Duration	0.909	1.100	<$10^{-7}$
	CoV Duration	0.767	1.798	0.171	Med. SPARC	0.842	1.642	<$10^{-3}$
	Med. SPARC.	0.755	2.627	0.017	Med. Max. Acc.	0.803	3.591	<$10^{-3}$
	CoV Max. Acc.	0.740	5.593	0.063	CoV Duration	0.770	1.719	0.194
**Comparison**	**PD OFF & Healthy Old**				**PD OFF & Healthy Young**			
	Med. Min. Accel.	0.892	1.332	<$10^{-7}$	Med. Min. Accel.	0.952	0.863	<$10^{-11}$
	Med. Duration	0.838	1.217	<$10^{-4}$	Med. Duration	0.918	0.916	<$10^{-8}$
	Med. SPARC	0.816	1.469	<$10^{-3}$	Med. SPARC	0.891	1.063	<$10^{-5}$
	CoV Duration	0.792	1.933	0.023	Med. Max. Acc.	0.801	3.283	<$10^{-3}$
	CoV Max. Accel.	0.751	1.975	0.069	CoV Duration	0.778	1.847	0.027
**Comparison**	**Healthy Young & Old**				**Healthy Young & Old [At-Home]**			
	Med. Duration	0.675	3.914	0.181	CoV Min. Acc.	0.871	1.608	<$10^{-7}$
	Med. Min. Accel.	0.656	2.658	0.181	Med. Min. Acc.	0.853	1.705	<$10^{-7}$
	Med. Max. Accel.	0.650	7.162	0.181	Med. Max Acc.	0.841	1.767	<$10^{-6}$
	Med. SPARC	0.637	4.392	0.223	CoV SPARC	0.834	1.334	<$10^{-6}$
	CoV Max. Accel.	0.591	7.198	0.313	Med. SPARC	0.689	3.487	0.077

^1^ Highest AUC.
